# Weight-cycling over 6 years is associated with pain, physical function and depression in the Osteoarthritis Initiative cohort

**DOI:** 10.1038/s41598-023-44052-3

**Published:** 2023-10-09

**Authors:** Heather K. Vincent, Alisa J. Johnson, Kim T. Sibille, Kevin R. Vincent, Yenisel Cruz-Almeida

**Affiliations:** 1 Department of Physical Medicine and Rehabilitation, University of Florida, PO Box 112730, Gainesville, FL 32608 USA; 2 Pain Research and Intervention Center of Excellence, University of Florida, Gainesville, FL USA; 3 Department of Community Dentistry and Behavioral Science, University of Florida, Gainesville, FL USA; 4 Translational Research in Assessment and Intervention Lab, University of Florida, Gainesville, FL USA; 5 Phenotyping and Assessment in Neuroscience Lab, University of Florida, Gainesville, FL USA; 6 Department of Neuroscience, University of Florida, Gainesville, FL USA; 7 Department of Epidemiology, University of Florida, Gainesville, FL USA

**Keywords:** Osteoarthritis, Outcomes research, Risk factors

## Abstract

Body weight significantly impacts health and quality of life, and is a leading risk factor for the development of knee osteoarthritis (OA). Weight cycling may have more negative health consequences compared to steady high or low weight. Using the Osteoarthritis Initiative dataset, we investigated the effects of weight cycling on physical function, quality of life, and depression over 72-months compared to stable or unidirectional body weight trajectories. Participants (n = 731) had knee OA and were classified as: (1) stable-low (BMI < 25), (2) stable-overweight (BMI = 25–29.9), and (3) stable-obese (BMI ≥ 30); (4) steady-weight-loss; (5) steady-weight-gain (weight loss/gain ≥ 2.2 kg every 2-years); (6) gain–loss–gain weight cycling, and (7) loss–gain–loss weight cycling (weight loss/gain with return to baseline), based on bi-annual assessments. We compared Knee Injury and Osteoarthritis Outcome Knee-Related Quality of Life, Function in Sports and Recreation, Physical Activity in the Elderly, Short Form SF-12, repeated chair rise, 20-m gait speed, and Center for Epidemiological Studies Depression using repeated-measures ANOVA. The steady weight loss group demonstrated the worst pain, physical function, and depressive symptoms over time (*p*’s < 0.05). More research is needed to confirm these findings, and elucidate the mechanisms by which steady weight loss is associated with functional decline in knee OA.

## Introduction

Long-term knee joint health, knee-related quality of life and overall wellbeing is dependent on maintenance of healthy body weight. Obesity is an independent risk factor for onset and progression of knee osteoarthritis (OA), impairment of knee function, and deterioration of physical and emotional wellbeing^1–3^. Functional biomarkers, such as timed stair climb and walking performance, are worse in individuals with higher obesity classes^4^. Cross-sectional data reveal worse self-efficacy with mobility^4^ and worse knee OA symptoms with higher body mass index (BMI) values than lower BMIs^5^. Perceived physical function and physical performance (e.g., walking and stair navigation) are both inversely related to depressive symptomology in obese persons with knee OA^6^. Machine learning models show that the five most important predictors of future mobility limitation were gait speed, chair rise performance, self-reported health status, BMI, and depression^7^.

Population trends show that people are now living longer with heavier body weight across the lifespan, and U.S. adults are now reporting progressively more attempts with weight loss than in years prior^8^. Weight loss occurring over months to years can improve knee pain severity, self-reported physical function, and mobility among individuals with knee OA^9^. Weight change trajectories are dynamic however, and can vary from stable, increasing, decreasing and cycling. Weight regain occurs in over 30% of individuals one year after weight loss and up to 95% by year five^10^. Weight cycling consists of variable magnitudes of weight gain and loss over time and places individuals at a greater risk for skeletal muscle mass loss, muscle strength decline, comorbid burden, onset of various cancers, and depressive symptoms^11–16^. While the role of body weight on knee OA symptoms is recognized, the effects of weight cycling on mobility loss, quality of life, and emotional wellbeing compared to stable weight or linear changes in body weight in this population, have not been previously examined. We conducted systematic literature searches and were not able to find investigations of these collective relationships up through May 2023. For patients with knee OA, disability risk prediction may become more precise with a better understanding of the potential impact of weight change patterns.

The purposes of this study were to determine among patients with knee OA: (1) the effect of body weight cycling within the context of BMI, on pain, physical function, function-related quality of life compared to stable and unidirectional weight/ BMI change, and (2) second, determine how the specific patterns of weight/ BMI change impacted depressive symptom scores over 72 months compared to stable or unidirectional patterns of body weight change. We hypothesized that progressive weight/BMI gain and weight/BMI cycling involving weight loss and regain, would both be associated with worse pain, physical function and quality of life outcomes, and higher depressive symptom scores compared to other weight and BMI patterns.

## Methods

### Study design

Data were obtained from the Osteoarthritis Initiative (OAI) clinical dataset version 0.2.3, release created 9.0401M4. These data are available for public access (https://nda.nih.gov/oai/) and permission was obtained by the team from NIMH Data Archive (NDA) to access the data. The OAI is a multicenter, observational population-based study of knee OA and is comprised of three subgroups (N = 4796): the Incidence Cohort, the Progression Cohort and the Control group. As originally designed, age-eligible women and men were recruited from the community and enrolled at four recruitment centers (University of Maryland Baltimore, Johns Hopkins University; Ohio State University; University of Pittsburgh; Memorial Hospital of Rhode Island/Brown University). The OAI was designed to better understand the progression of knee OA and treatment impact in people at-risk-for or with the disease. All participants underwent comprehensive clinical screenings including radiographic imaging and surveys. Kellgren Lawrence (KL) osteoarthritis severity scores were applied by radiologists at the participating sites.

### Participants and grouping

Participants were first classified into seven groups based on temporal change patterns of body weight assessed biannually, over a 72-month period (N = 731). The definition of weight cycling was applied from the Diabetes Prevention Program Research Group^17^. Cycling was defined as body weight change by ≥ 2.2 kg with return to at least that same body weight two years later. The groups were then converted to body mass index (BMI) categories for clinical context. We acknowledge that there are many additional weight/BMI change patterns that existed. Given the sheer volume of data, we present here two common weight cycling patterns in addition to stable and unidirectional weight change. Therefore, the seven groups were as follows:

1. Stable-low-weight (BMI < 25 kg/m^2^) weight at each time point 63.0 ± 9.6 kg
2. Stable-overweight (BMI = 25–29.9 kg/m^2^) weight at each time point 77.2 ± 9.8 kg
3. Stable-obese (BMI ≥ 30 kg/m^2^) weight at each time point 92.0 ± 12.4 kg
4. Steady-weight-loss (loss ≥ 2.2 kg every 2-years) weight at each time point: 90.1 ± 11.8 kg, 85.7 ± 11.6 kg, 81.2 ± 12.2 kg and 76.2 ± 13.2 kg,
5. Steady-weight-gain (gain ≥ 2.2 kg every 2-years) weight at each time point: 89.1 ± 14.4 kg, 95.2 ± 15.6 kg, 99.9 ± 15.6 kg and 104.7 ± 17.1 kg
6. Gain–loss–gain weight cycling (gain ≥ 2.2 kg, loss ≥ 2.2 kg, gain ≥ 2.2 kg) weight at each time point: 84.7 ± 16.2 kg, 90.9 ± 16.3, 83.1 ± 15.2 kg and 88.8 ± 16.4 kg
7. Loss–gain–loss weight cycling (loss ≥ 2.2 kg, gain ≥ 2.2 kg, loss ≥ 2.2 kg) weight at each time point: 91.0 ± 13.9 kg, 84.8 ± 12.9, 90.4 ± 13.6 kg and 85.1 ± 13.6 kg.

**Figure 1:**
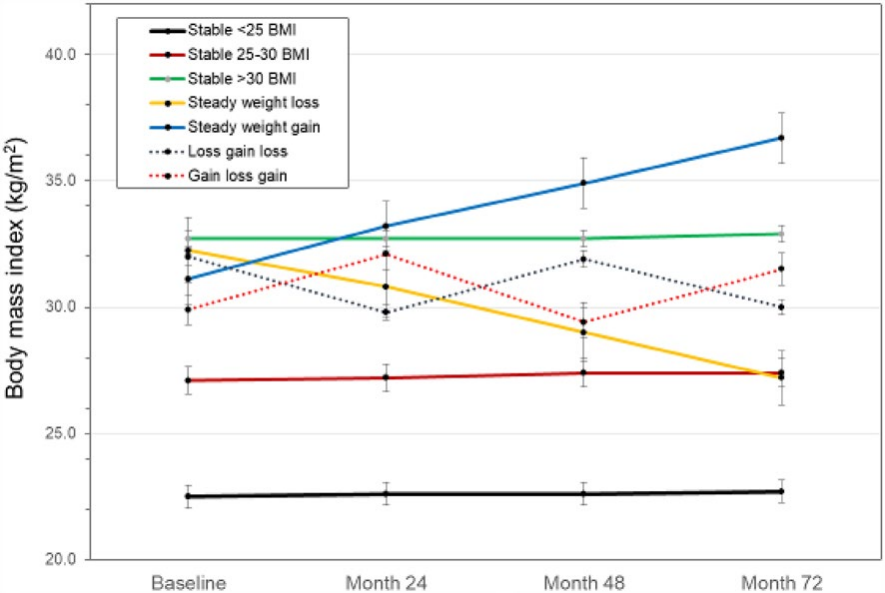
BMI change patterns over time. Values are means ± SD.

To be included in the analyses, participants had to have body weight and BMI values at each of the four time points with the minimum threshold for change defined above. Participants who underwent a total knee or total hip replacement were also removed from the dataset to eliminate the confounding effect of surgery-induced improvement in function and pain over six years. Data from the remaining 731 participants were included in these analyses. Figure 1 provides the BMI patterns among the seven participant groups. These data demonstrate the successful separation of participants into the weight change strata outlined here.

### Health status and covariates at baseline

Anthropometrics, demographics, and cigarette smoking status were extracted from the dataset. Body mass index (BMI) was calculated as weight (kg) divided by the square of height (m^2^). The age-adjusted Charlson Comorbidity Index (CCI)^18^ was calculated as a comorbid burden estimate, where age adjustment consisted of assigning each decade of life a comorbidity score of one point in addition to presence or not of 19 conditions. Several characteristics were extracted from the datasets that could influence study outcomes and were covariates. These included: (1) insurance status (some form of health insurance or not), (2) marital status (married or not), (3) educational status (stratified into less than high school or not), (4) employment (currently working for pay or not), (5) household income (< or ≥ $50,000), and (6) home living situation (living alone or not). Kellgren Lawrence (KL) osteoarthritis severity scores^19^ for both knees were extracted from the dataset from available patients. The use of pain medications was extracted from the question *did you take pain medications today* (yes/no).

### Knee pain severity and total painful joints

Presence of knee pain was considered a secondary outcome and was defined as the participant report of *pain, aching, or stiffness on more than half days a month, past 12 months*^20^, Pain severity was reported in the dataset using an 11-point numerical pain rating scale, where 0 = no pain and 10-worst possible pain. Pain presence and severity were collected at baseline and month 72. We determined the prevalence of knee joints that experienced no pain (pain-free, with scores of 0) at baseline and developed pain at month 72. The prevalence of participants who had knee pain at baseline (score ≥ 1 point) who did not report knee pain at month 72 (pain-free) was also determined. The total number of lower body painful joints was summed from right and left foot, ankle, knee, hip and the back at baseline and month 72.

### Subjective outcome measures

Five self-reported and performance-based functional metrics were selected to represent functional capacity (in the home, work, community and social domains), and quality of life. These included the Knee Injury and Osteoarthritis Outcome Score (KOOS), Short Form SF-12, Physical Activity in the Elderly (PASE), repeated chair rise test and the 20-m gait speed test.

#### Knee injury and osteoarthritis outcome score (KOOS)

The KOOS evaluates the short and long-term symptoms in individuals with knee osteoarthritis or injury^21^. This is a valid and reliable instrument that consists of five different subscales, two of which were included: Function in Sports and Recreation (KOOS-SFR; involves questions about difficulty with tasks such as jumping, running, squatting or kneeling), and the knee-related quality of life (KOOS-QOL; involves questions about awareness of knee problems, modifications to lifestyle). From Likert scale responses on SFR and QOL items, scores are transformed to a 0–100 scale with 0 representing extreme knee problems and 100 representing no knee problems^22^. The KOOS has good internal consistency and test–retest reliability^23^, correlates with other self-reported measures of physical function and is sensitive to change^24,25^.

#### Short form SF-12

The SF-12 is a generic QOL instrument that captures self-reported impact of health on everyday life and is a shorter version of the SF-36^26^. Eight health-related domains with one or two questions per domain are included (limitations in physical activities, social activities and usual role activities, bodily pain, general mental health, vitality and general health perceptions). A Physical Composite Score (PCS) was determined using scores from the 12 questions. The scores are norm-based, with a mean of 50 in the general population, and higher scores indicate better health^26^. The SF-12 is validated for use in osteoarthritis and has strong correlations with clinical variables^27^.

#### Physical activity in the elderly (PASE)

The PASE is a valid, 12-item self-administered questionnaire developed for the use in adults over 65 years of age to estimate the amount of physical activity performed over the last seven days^28^. Physical activities include walking outside, household chores (light or heavy), sports and recreational activities (light, moderate and strenuous, muscle strengthening/endurance activity) and work hours. The intensity level, duration and frequency are used to create a score ranging from 0 to 793 points, with higher scores representing higher activity levels^28^. This instrument has good test–retest reliability (r = 0.75), and has recently been recommended as the assessment to measure total physical activity in older adults^29^. Subscales for muscle strengthening/endurance activity and walking activity in hours per day were used.

### Physical function tests

Performance-based, valid measures of physical function extracted from the dataset included the repeated chair rise test (timed completion of five chair stands), and the 20-m gait speed test. Gait speeds and gait speed loss are related to loss of independence and quality of life^30^. Gait speed was determined by dividing the time to complete the test by the walk test distance, and are expressed in m/s. Loss of mobility was estimated using gait speed thresholds and reduction in gait speed over time. Gait speed of less than 1 m/s is associated with elevated risk for self-reported mobility disability among older adults^31^. A loss in gait speed of > 0.2 m/s is associated with an elevated risk for mortality among individuals with knee OA^32^. Mobility loss in this study was operationalized as the change in gait speed from baseline to month 72. The proportion of individuals in each BMI group with mobility loss was calculated for analysis.

### Center for epidemiological studies depression score (CES-D)

The CES-D is a 20-item instrument that asks respondents to rate how often over the past seven days they experienced symptoms associated with depression, such as trouble focusing, poor appetite, and feelings of loneliness, sadness, failure in life self-dislike^33^. Response options range from 0 to 3 for each item (0 = *Rarely or None of the Time*, 1 = *Some or Little of the Time*, 2 = *Moderately or Much of the time*, 3 = *Most or Almost All the Time*). Scores range from 0 to 60, with high scores indicating greater depressive symptoms. The CES-D has high reliability in older adults (Cronbach’s alpha 0.84)^34^, and can effectively discriminate between persons with and without depression who have chronic pain (sensitivity 81.8%, specificity 72.7%)^35^.

### Statistical analyses

Data analysis was conducted in IBM SPSS version 26.0 (Armonk, NY). Distributional form was examined for each outcome variable. For outcomes where skewness and kurtosis existed (i.e., 20-m gait speed, repeated chair rise time, and SF-12 Physical subscore), Log10 transformations were performed. Given that the analyses of the transformed variables yielded the same results as the raw data, we present the raw data here for ease of interpretation. Kruskal–Wallis tests were performed on baseline categorical variables, presence of joint pain at baseline and month 72, and changes in mobility (yes/no values for walking speed of < 1 m/s and a reduction in gait speed over time by > 0.2 m/s). Bonferroni corrections at an alpha level of 0.05 were used to adjust for Type 1 errors in the Kruskal–Wallis tests. One-way analyses of variance (ANOVAs) were used to determine if BMI group differences existed in continuous baseline variables. Repeated measures ANOVAs were used to determine BMI group differences on clinical outcomes (pain, function, quality of life, depressive symptoms), and KL scores. Based on baseline BMI group differences sex, age, race, marital status, employment, education level, CCI, number of painful joint pain sites and pain medication use were included as covariates in the analyses. Univariate ANOVA was performed on change in gait speed from baseline to month 72, adjusted for baseline speed. Participants with complete datasets for each outcome variable were included in each analysis. Statistical significance was set at *p* < 0.05.

## Results

### Participant characteristics

Characteristics at baseline are presented in Table 1. BMI group differences were found for weight and waist (*p*’s < 0.001). The mean percent fluctuation ranges in body weight over two-year intervals were less than 0.5% for all stable BMI groups (i.e., BMI < 25 kg/m^2^, BMI 25–29.9 kg/m^2^, BMI ≥ 30 kg/m^2^), − 4.9% to − 6.4% in the steady-weight, 5.0% to 6.9% in the steady-weight-gain group, − 5.9% to 6.6% in the loss–gain–loss group, and 7.6% to − 8.4% in the gain–loss–gain group. Several other BMI group differences existed in age, sex, marital status, employment, education, CCI, KL score, total number of painful joints and pain medication use. The steady-weight-loss group had the highest percentage of participants who were female, unmarried, not employed and had the highest CCI scores, KL scores, and number of painful joints (all *p* < 0.05).

**Table 1:** Baseline characteristics (N = 730).

BMI	Stable	Stable	Stable	Steady	Steady	Cycling	Cycling	
	< 25 kg/m^2^	25–29.9 kg/m^2^	> 30 kg/m^2^	Weight loss	Weight gain	Loss gain loss	Gain loss gain	
(n)	(220)	(206)	(97)	(24)	(24)	(85)	(75)	*p*
Age (yr)	61.2 ± 9.3	63.3 ± 9.0	60.1 ± 8.5	62.7 ± 9.3	55.3 ± 7.0	60.4 ± 9.0	57.9 ± 8.2	< 0.001
Weight (kg)	62.9 ± 9.4	76.9 ± 9.7	92.0 ± 12.3	90.1 ± 11.8	89.2 ± 14.4	91.0 ± 13.9	84.7 ± 16.2	< 0.001
Waist (cm)	87.8 ± 7.9	98.7 ± 7.6	109.9 ± 9.6	111.1 ± 9.4	111.5 ± 11.6	109.3 ± 11.2	105.9 ± 11.7	< 0.001
Sex # (% F)	146 (66.4)	104 (50.5)	52 (53.6)	17 (70.8)	14 (58.3)	45 (52.9)	49 (65.3)	0.014
Race # (%)								
African American	40 (18.2)	36 (17.5)	24 (24.7)	6 (25.0)	7 (29.2)	14 (16.5)	12 (16.0)	
Caucasian	173 (78.6)	166 (80.6)	69 (71.2)	17 (70.8)	17 (70.8)	69 (81.2)	60 (80.0)	
Other	7 (3.2)	4 (1.9)	3 (3.1)	1 (4.2)	0 (0.0)	2 (2.4)	3 (4.0)	0.135
Ethnicity # (% Hispanic)	4 (1.8)	2 (0.9)	1 (1.0)	0 (0.0)	0 (0.0)	0 (0.0)	2 (2.7)	0.720
Marital status # (% married)	162 (73.6)	157 (76.2)	63 (64.9)	13 (54.2)	16 (69.6)	51 (60.7)	47 (63.5)	0.015
Living alone # (%)	52 (24.0)	42 (20.5)	17 (17.7)	8 (33.3)	5 (21.7)	27 (31.8)	17 (22.7)	0.715
Currently employed # (%)	150 (68.2)	115 (55.8)	67 (69.1)	14 (58.3)	20 (83.3)	57 (67.1)	52 (69.3)	0.011
Income < $50 K # (%)	75 (34.1)	68 (3.3)	39 (40.2)	12 (50.0)	7 (29.2)	30 (35.3)	29 (38.7)	0.679
Education, < HS # (%)	6 (2.7)	6 (2.9)	3 (3.1)	1 (4.2)	1 (4.2)	3 (3.6)	3 (4.0)	0.011
Insurance status # (% insured)	193 (89.8)	183 (90.1)	85 (89.5)	21 (87.5)	21 (91.3)	72 (84.7)	64 (86.5)	0.852
Smoking now # (%)	5 (2.3)	7 (3.4)	3 (3.1)	2 (8.3)	2 (8.3)	5 (5.9)	5 (6.7)	0.352
CCI (points)	2.0 ± 1.4	2.1 ± 1.2	1.7 ± 1.0	2.3 ± 1.4	1.1 ± 0.9	1.9 ± 1.3	1.2 ± 0.9	< .001
Total Painful joints	2.0 ± 1.6	2.2 ± 1.8	2.4 ± 1.6	2.5 ± 1.7	2.9 ± 2.2	2.6 ± 1.9	2.7 ± 1.8	0.010
Pain medication use # (% yes)	13 (5.9)	15 (7.4)	10 (10.4)	4 (16.7)	1 (4.2)	15 (17.6)	17 (23.0)	< 0.001

alues are means ± SD or % of the group.

*BMI* Body mass index; *HS* High school; *CCI* Charlson Comorbidity Index; *KL* Kellgren Lawrence.

Supplemental Table 1 provides the severity of knee pain for each group at baseline and month 72. Group differences existed among the pain severity for both limbs at baseline and at month 72; the highest left and right knee pain ratings in the steady weight loss and loss–gain–loss groups (*p* < 0.001). The *prevalence* of participants who became pain free by month 72 was lowest in the steady weight gain and steady weight loss groups (0–11%), and highest in the stable weight > 30 kg/m^2^ (left limb) and stable weight 25–29.9 kg/m^2^ (right limb). The highest prevalence of pain free joints that became painful by month 72 were in the gain–loss–gain group (left limb) and steady weight gain group (80.0%; right limb). Supplemental Fig. 1 shows the change in KL scores from baseline to month 72 using available data from each BMI group. A group X time interaction was found for KL score in the right knee (*p* = 0.038), but not the left.

### Physical function outcomes

**Figure 2:**
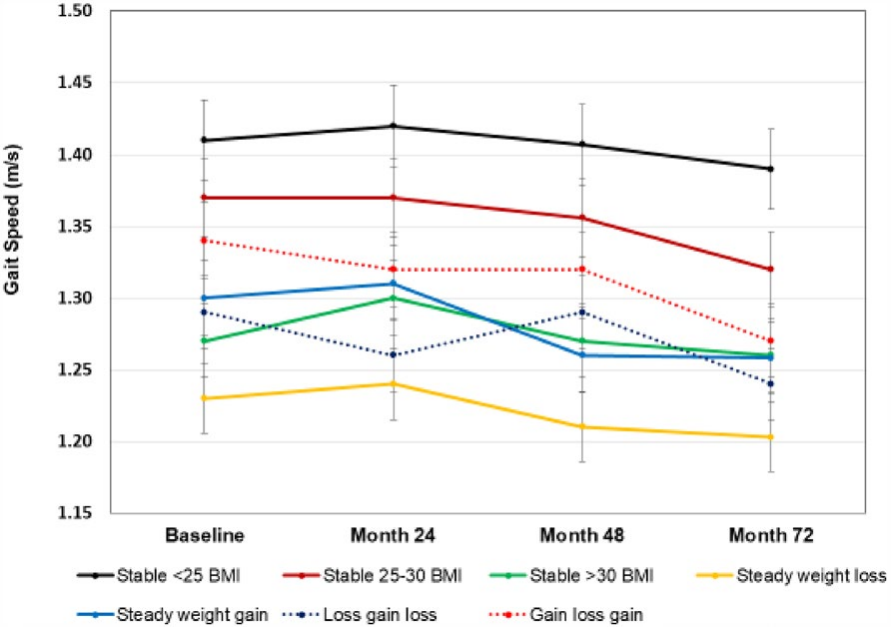
Gait speed over time by weight change groups.Values are means ± SD.

**Figure 3:**
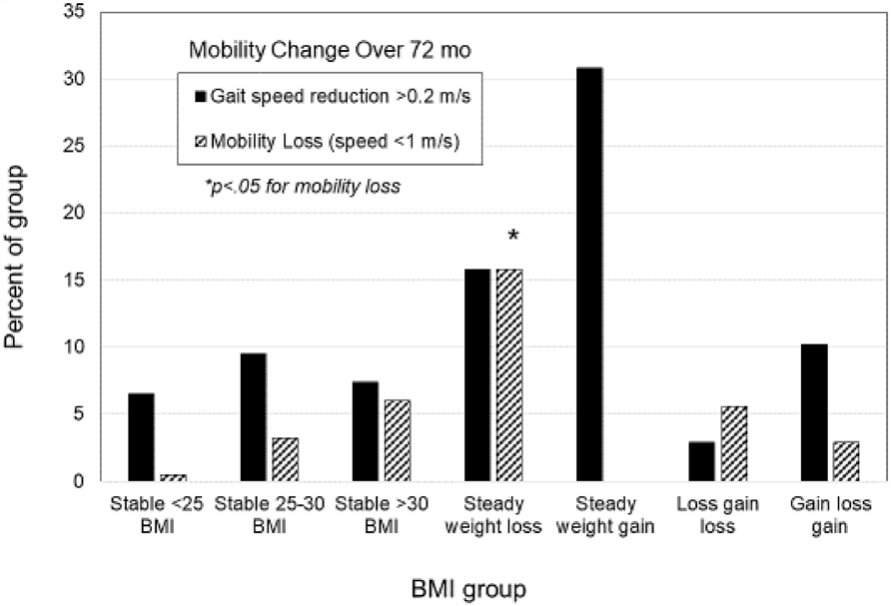
Change in mobility over time as defined by a decrease in gait speed of > 0.2 m/s and by habiutal walking speed of < 1 m/s. Values are percent of the group. *Denotes different from stable-low < 25 BMI group at *p* < 0.05.

**Figure 4:**
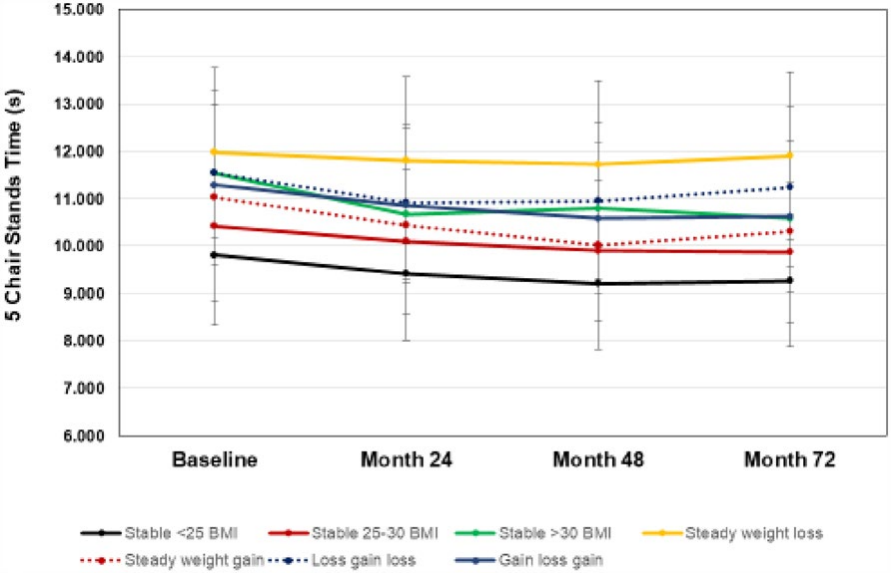
Completion time of five chair stands over time by weight change groups. Values are means ± SD.

Performance-based measures of function are presented in Figs. 2, 3, and 4. The interaction for group X time was statistically significant for gait speed (*F*(4,18) = 1.55, *p* = 0.002), and for the main effect of group on gait speed (*p* < 0.001). For changes in mobility, a greater percentage of participants in the steady-weight-loss group experienced mobility loss (habitual gait speeds < 1 m/s) over the study period compared to the stable-low BMI group (*p* = 0.008). As seen in Fig. 4, there was no statitistically significant group X time interaction for repeated chair stand time (*p* = 0.929), but a main effects for BMI group were statistically significant (*p* < 0.001). For both gait speed and chair rise tests, the steady-weight-loss group performed the worst over time.

**Figure 5:**
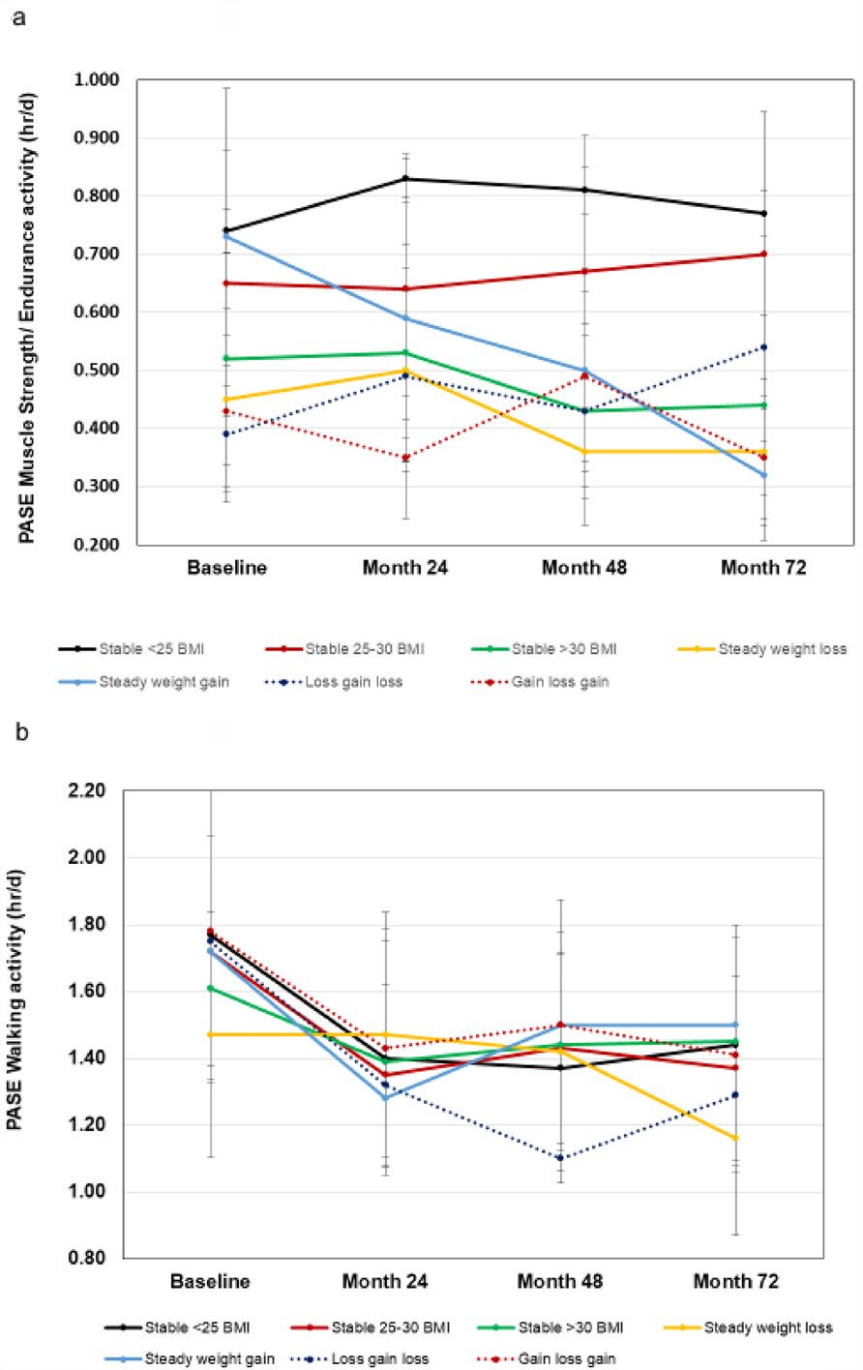
Physical Activity Scale for the Elderly (PASE) subscores for daily participation in muscle strengthening or endurance exercise and walking activity subscores in different BMI trajectories. Values are means ± SD.

The self-reported measures of physical function, KOOS and PASE scores are provided in Table 2 and Fig. 5a, b. The group X time interaction for KOOS-QOL was found to be statistically significant (*F*(4,18) = 3.045, *p* < 0.001), with significant main effects of BMI group and time (*p*’s < 0.001). Group X time interaction effect was not statistically significant for KOOS SFR, but there was a main effect for BMI group (*p* < 0.001), such that the steady-weight-loss group reported the lowest SFR scores over time. The group X time interaction was statistically significant for SF-12 PCS scores (F(4,18) = 1.855 *p* = 0.016), with main effects for group and time (*p*’s < 0.05). There was no statistically significant group X time interaction for PASE muscle strengthening activity (*p* = 0.361), but there was a main effect for group (*p* < 0.001). There was no statistically significant interaction or main effects of group or time found for PASE daily hours of walking (*p* = 0.562).

**Table 2:** Subjective functional scores over six years with different patterns of weight change.

BMI	Stable weight	Stable weight	Stable weight	Steady	Steady	Cycling	Cycling
	< 25 kg/m^2^	25–29.9 kg/m^2^	> 30 kg/m^2^	Weight loss	Weight gain	Loss gain loss	Gain loss gain
(n)	(220)	(205)	(95)	(24)	(24)	(83)	(74)
KOOS QOL (points)							
Baseline	76.3 ± 19.8	73.1 ± 20.6	67.9 ± 19.5	59.9 ± 22.6	64.9 ± 21.6	66.5 ± 23.2	66.9 ± 23.0
24 mo	80.3 ± 17.3	75.4 ± 20.0	74.9 ± 17.9	62.0 ± 21.1	68.5 ± 21.0	67.4 ± 22.2	67.2 ± 23.3
48 mo	79.9 ± 18.5	79.3 ± 19.6	71.9 ± 19.7	65.6 ± 22.1	64.4 ± 23.7	66.6 ± 21.9	74.0 ± 20.6
72 mo	79.3 ± 19.6	78.6 ± 19.9	71.9 ± 20.2	65.1 ± 20.0	57.9 ± 24.1	65.0 ± 20.4	68.3 ± 21.7
KOOS SFR (points)							
Baseline	85.3 ± 17.9	82.0 ± 21.4	77.5 ± 19.6	61.6 ± 28.8	76.7 ± 32.1	74.3 ± 27.8	76.6 ± 22.4
24 mo	87.0 ± 17.9	84.0 ± 21.5	79.0 ± 20.0	60.2 ± 27.3	75.2 ± 28.7	75.7 ± 24.3	79.0 ± 19.6
48 mo	87.1 ± 19.0	85.7 ± 19.7	81.0 ± 19.1	54.4 ± 27.8	75.8 ± 27.9	70.6 ± 25.6	81.6 ± 21.0
72 mo	85.2 ± 19.4	85.0 ± 20.1	75.8 ± 23.2	61.1 ± 16.9	70.2 ± 34.8	74.3 ± 24.7	77.9 ± 22.2

BMI	Stable weight	Stable weight	Stable weight	Steady	Steady	Cycling	Cycling
	< 25 kg/m^2^	25–29.9 kg/m^2^	> 30 kg/m^2^	Weight loss	Weight gain	Loss gain loss	Gain loss gain
(n)	(212)	(195)	(92)	(22)	(22)	(76)	(71)
SF-12 PCS (points)							
Baseline	53.3 ± 6.5	51.2 ± 7.8	49.5 ± 8.8	45.6 ± 8.8	47.1 ± 11.5	48.4 ± 9.6	47.6 ± 9.8
24 mo	53.4 ± 6.1	50.7 ± 7.9	49.1 ± 7.9	44.9 ± 9.7	50.3 ± 10.8	47.5 ± 10.8	47.5 ± 9.2
48 mo	52.2 ± 6.4	50.7 ± 7.8	48.0 ± 8.8	47.1 ± 9.6	45.3 ± 10.5	47.1 ± 9.5	47.2 ± 11.0
72 mo	52.3 ± 6.8	49.7 ± 8.9	47.0 ± 8.2	45.3 ± 10.4	44.3 ± 12.7	45.1 ± 10.4	45.1 ± 10.1

KOOS = Knee Osteoarthritis Outcome Score; SF-12 PCS = Medical Outcomes Short Form-12, Physical Component Score. KOOS-QOL and SF-12 PCS are shown at 24-month intervals by BMI group. Values are means ± SD. For KOOS-QOL and SF-12 PCS, group x time interactions and main effects of group and time were significant at *p* < 0.05. KOOS-SFR, only a main effect of group existed at *p* < 0.05.

### Depressive symptomatology

CES-D scores are provided in Table 3. The group X time interaction did not achieve statistical significance (*F*(4,18) = 1.453, *p* = 0.098), but there was main effect of group (*p* < 0.001). The steady-weight-loss group reported the highest scores at each time point.

**Table 3:** Center for Epidemiological Studies Depression Score (CES-D) over six years with different patterns of weight change.

BMI	Stable weight	Stable weight	Stable weight	Steady	Steady	Cycling	Cycling
	< 25 kg/m^2^	25–29.9 kg/m^2^	> 30 kg/m^2^	Weight loss	Weight gain	Loss gain loss	Gain loss gain
(n)	(214)	(201)	(95)	(22)	(23)	(78)	(73)
CES-D (points)							
Baseline	4.3 ± 5.8	4.1 ± 4.6	5.5 ± 6.5	11.1 ± 11.76	5.7 ± 6.5	7.4 ± 8.5	6.5 ± 6.8
24 mo	4.8 ± 4.9	4.9 ± 5.6	6.1 ± 6.4	9.6 ± 7.6	5.4 ± 5.7	8.4 ± 9.8	5.9 ± 6.8
48 mo	5.4 ± 5.8	4.8 ± 5.4	6.0 ± 5.7	13.8 ± 10.2	7.7 ± 7.7	8.1 ± 8.4	7.4 ± 8.1
72 mo	5.4 ± 5.8	4.8 ± 5.4	6.4 ± 6.8	13.6 ± 10.6	7.8 ± 10.2	8.7 ± 9.2	8.1 ± 7.2

Scores shown at 24-month intervals by BMI group. Values are means ± SD. A main effect of group (*p* < 0.001) existed.

## Discussion

Our purpose was to determine the effect of body weight cycling on self-reported and performance-based measures of pain, physical function, quality of life (QOL), and depressive symptomatology compared to stable or unidirectional trajectories of body weight in persons with knee osteoarthritis (OA). In contrast to our expectations, patients with steady weight loss over 72 months emerged as the group with the worst pain and physical function, greater mobility loss, and worst QOL. Patients with stable weight patterns (i.e., BMI < 25 kg/m^2^ and 25–30 kg/m^2^) reported consistent daily participation in muscle strengthening/endurance activity and low depressive symptoms over time, commensurate with preservation of gait speed and chair stand time, as well as more positive self-reported outcomes (i.e., pain, KOOS, and SF-12 scores). Weight cycling patterns of ±  ≥ 2.2 kg did not have a significant effect on any study outcome.

The main findings were in contrast to our hypotheses. We did demonstrate the expected patterns of generally worse outcomes with stable, but BMI > 30 kg/m^2^ trajectories and with linear weight/ BMI gain over time compared to stable BMI < 25 kg/m^2^ at each timepoint. In this study, we grouped participants in weight/BMI cycling groups if they demonstrated a minimum weight change of 2.2 kg over two years. It is possible that larger group differences in subjective and objective outcomes may have emerged with a larger weight change threshold. Another possibility is that weight cycling may change physical function reserves, depending on the initial body weight. For example, weight loss in a person with high BMI may improve functional outcomes, but have limited or worsening effect among individuals with healthy BMI values. Over time, potential effects of the same weight change may wash out functional effects when averaged across one weight/BMI trajectory. This area warrants additional study to determine the precise impact of weight gain or loss among different BMI strata. The reason for the worse outcomes in the steady weight loss group are not clear. The data show that this group experienced the highest depressive symptom burden even at baseline (Table 3) and a high prevalence of living alone, not married and the highest prevalence of low income compared to most groups. The Charlson comorbid index on average was also highest among all groups. This ‘profile’ may represent a group who might not want to, or cannot, be as functionally capable if they do not have the support or mental wellbeing to do so. Our upcoming research is working to understand the prospective relationship between psychological stress, BMI and physical function.

Comparative studies of weight cycling effects on clinical outcomes in this population are very limited, and research has focused primarily on the structural progression of knee OA with linear changes in body weight and subjective function. Compared to stable weight, progressive weight gain is related to worsening cartilage defects using magnetic resonance imaging data and worsening synovitis^36,37^, whereas weight loss reduces medial tibial cartilage loss and improves knee symptoms and subjective function^38^. Prior studies using OAI data showed that participants who lost > 10% body weight compared to 5–10% body weight had less progression of cartilage deterioration over four years than participants with stable weight^39^. There appears to be a dose–response relationship of body weight change on pain and function outcomes. For example, weight gain (> 10%) is associated with increased self-reported pain and decreased physical function scores compared to smaller body weight changes (< 5%)^40^. Evidence from studies in older adult cohorts have reported relationships between weight cycling and mobility disability, such that the risk for self-reported mobility disability was elevated in patients who had lost weight or cycled their body weight by 5% compared to patients with stable weight (hazard ratios ranged from 1.39 to 1.67)^41^. In the Look AHEAD Study, individuals with type II diabetes were examined over 8–9 years. Female weight cyclers demonstrated worse physical performance (9–10% worse Physical Performance Battery scores, 6–8% slower gait speed) than weight losers and maintainers. Males produced 8–19% lower handgrip strength scores than weight re-gainers or weight losers and maintainers^42^.

Our primary study finding, that steady weight loss was associated with worse outcomes, highlights an important consideration for older adults. Clinical relevance of the differences among BMI groups should be with caution. Some objective differences in gait speed (ranging from 2 to 13%; 0.03–0.18 m/sec) and chair rise time (ranging from 4 to 18%; 0.4–2.7 s) were small but statistically significant. Similarly, subjective group differences among KOOS (ranging from 4 to 28%; 3.3–24.1 points) and SF-12 scores (ranging from 4 to 15%; 1.5–7.9 points) were also statistically significant. Minimal clinically important differences (MCID) the repeated chair rise time is 2.3 s and in gait speed 0.2 m/s. MCID for SF-12 PCS are 1.8 points, and for the KOOS 19.6–21.1 points among persons with severe OA and knee replacement. Thus, our data generally fall close to these values. Muscle loss may accompany overall body weight loss and thereby impede physical function. The impact of steady weight loss in middle-to-older age individuals with knee OA may be counteracted with consistent participation in muscle strengthening and endurance activities. Unfortunately, we were not able to ascertain the loss of muscle mass versus adipose tissue in our groups thus limiting the conclusions that can be drawn from these findings. Future work in this area is needed to better understand the factors contributing to functional decrements in this population.

Interestingly, we found that those who *perceived* greater difficultly with completing daily activities and considered themselves more functionally limited walked and rose from a chair more slowly than participants who self-rated higher function. Participants who *perceived* less disability and functional impairment demonstrated better physical performance scores over time. This differential response among groups is likely influenced by factors in addition to body weight. These factors could include variations in confidence in knee function^43^, knee pain severity^44^, pain-related cognitions such as fear, self-efficacy^45^ and catastrophizing^46^, other psychological factors^47,48^, and comorbid disease burden. Low levels of knee confidence, negative emotional states (depression), and unfavorable psychological profiles (‘severe’, ‘extremely troubled’) are related to higher self-rated disability, worse walk speeds, chair stand time and stair navigation, and elevated pain^6,43,47^. Depressive symptoms are closely interrelated with physical disability^49^, and depression accelerates OA progression^50^. Elevation of depressive symptomatology over time correspond with higher incidence of mobility disability among older adults^49^. This is also supported by our own findings where self-reported depressive symptomatology was highest and lowest among the steady weight loss and the stable weight groups, respectively. Among patients with knee OA and obesity, depression is related to perceived impairment of function and less to measured physical task performance^6^. Thus, self-reported difficulties in function may have more to do with emotional burden rather than physical difficulty alone. The interrelationships of these factors are complex, affecting patient beliefs and subsequently, functional outcomes and quality of life^48^. Comorbid obesity and depression are independently associated with functional limitations, but have not been shown to independently modify the relationship between knee OA and physical function^51^. Pain severity and patient beliefs also influence physical function in patients with OA^51^. Fear of movement is common in patients with knee OA and commonly results in avoidance of physical activities that could hurt while performing them^52^, which further contributes to loss of muscle strength and endurance^51^. Additional work is needed to determine the relationships between dynamic changes in body weight and composition, physical activity, psychological characteristics, and pain and disability in this population.

Among adults with OA and obesity, lower limb strength predicts walking and weight-bearing task performance^44^, while 60% of the variance in self-reported physical function can be explained by pain severity, BMI, anxiety and leg muscle weakness^53^. Muscle strengthening and endurance benefits for clinical management of knee OA have been systematically reviewed and meta-analyzed, and indicate improvements in muscle strength, pain and disability occur^54^. Inclusion of regular exercise also reduces depression symptoms^55^, manages numerous comorbidities^56^, mitigates muscle loss^57^ and reduces activity limitations^58^. Individuals with knee OA may derive significant physical and psychological long-term benefit from participation in some type of muscle strengthening/ endurance activity, especially when coping with depressive symptoms^59^.

### Limitations and future directions

We had the opportunity to leverage the robust OAI dataset with clinically valuable measures, but there are some limitations to this study that deserve comment. A significant challenge in the study of weight cycling overall is the inconsistency on the agreement of a definition of what weight cycling is^11^. Clear definitions and discrete categories of weight change in both absolute and relative terms will improve interpretability of findings in future work. While we examined the impact of weight and BMI trajectories on our outcomes, detailed information of body composition (adipose vs. skeletal muscle), specific leg strength changes, in addition to information on intentional weight loss strategies could have clarified some of our observations on performance-based metrics. A limitation of many large population studies is the heavy use of self-report measures. In the OAI, several validated, reliable, but self-report measures related to pain effects on quality of life and physical function were used. These measures likely comprise elements of recall, social desirability or habituation bias. As the PASE instrument was designed for the elderly, but the OAI included adults as young as 55 years, there may be some items that are less relevant for the middle-aged participants. We acknowledge that depression is only one aspect of mental health and future research focused on disentangling the unique impact of various combinations of psychological and cognitive factors relevant to OA-related pain severity, specific comorbid conditions, and weight trajectories is needed. Expansion of psychological factors such as measures of fear, confidence, self-efficacy, and multivariable psychological profiles in future prospective studies of OA would greatly improve our interpretation of pain and functional changes over time, and guide the development of precise interventions to prevent disability in this population. We acknowledge that a couple of our study groups had small samples, and that multiple comparisons were made across several outcomes, both of which may have affected our results and produced some findings that may have been due to chance. Finally, the lack of complete data for all participants limited our sample sizes in some trajectory groups for six-year follow-ups; it is possible that there may be additional weight trajectory effects that were masked due to small samples. In light of these limitations, the generalizability to the OA population as a whole may not yet be possible. Prospective datasets that provide annual or biannual assessment of body weight, BMI in conjunction with physical function and mental health over 30–40 years could provide more insight on the small to large weight cycling impact that is more generalizable.

## Conclusions

In this cohort of middle to older-aged adults with knee OA, we did not detect significantly worse effects of weight cycling (gain–loss–gain or loss–gain–loss) on functional outcomes, quality of life or depressive symptoms compared to stable or unidirectional weight change trajectories. Steady weight loss was associated with worse outcomes and highest depressive symptomatology over a 72-month follow-up compared to stable weight, unidirectional or the two weight cycling patterns studied here. Consistent participation in muscle strengthening activities, especially among individuals with steady weight loss alone or in combination with strategies to improve psychological wellbeing may help counteract functional decline.

### Supplementary Information

Supplementary Figure 1.

Supplementary Table 1.

## Data Availability

Data were obtained from the Osteoarthritis Initiative (OAI) clinical dataset version 0.2.3, release created 9.0401M4. These data are available for public access (https://nda.nih.gov/oai/) and permission was obtained by the team from NIMH Data Archive (NDA) to access the data.
