# Author Correction: Weight-cycling over 6 years is associated with pain, physical function and depression in the Osteoarthritis Initiative cohort

**DOI:** 10.1038/s41598-024-52366-z

**Published:** 2024-01-19

**Authors:** Heather K. Vincent, Alisa J. Johnson, Kim T. Sibille, Kevin R. Vincent, Yenisel Cruz-Almeida

**Affiliations:** 1 Department of Physical Medicine and Rehabilitation, University of Florida, PO Box 112730, Gainesville, FL 32608 USA; 2 Pain Research and Intervention Center of Excellence, University of Florida, Gainesville, FL USA; 3 Department of Community Dentistry and Behavioral Science, University of Florida, Gainesville, FL USA; 4 Translational Research in Assessment and Intervention Lab, University of Florida, Gainesville, FL USA; 5 Phenotyping and Assessment in Neuroscience Lab, University of Florida, Gainesville, FL USA; 6 Department of Neuroscience, University of Florida, Gainesville, FL USA; 7 Department of Epidemiology, University of Florida, Gainesville, FL USA

Correction to: *Scientific Reports* 10.1038/s41598-023-44052-3, published online 09 October 2023

The original version of this Article contained an error in the name of Yenisel Cruz-Almeida, which was incorrectly given as Yenisel Almeida-Cruz.

The original Article has been corrected.

