# Supplementary material for: Weight-cycling over 6 years is associated with pain, physical function and depression in the Osteoarthritis Initiative cohort
Source: Sci Rep. 2023 Oct 9;13:17045. doi: 10.1038/s41598-023-44052-3 (PMC10562481; doi:10.1038/s41598-023-44052-3)
Supplement: Supplementary file 1 — Supplementary Figure 1. [file 41598_2023_44052_MOESM1_ESM.pdf]

Supplemental Figure 1

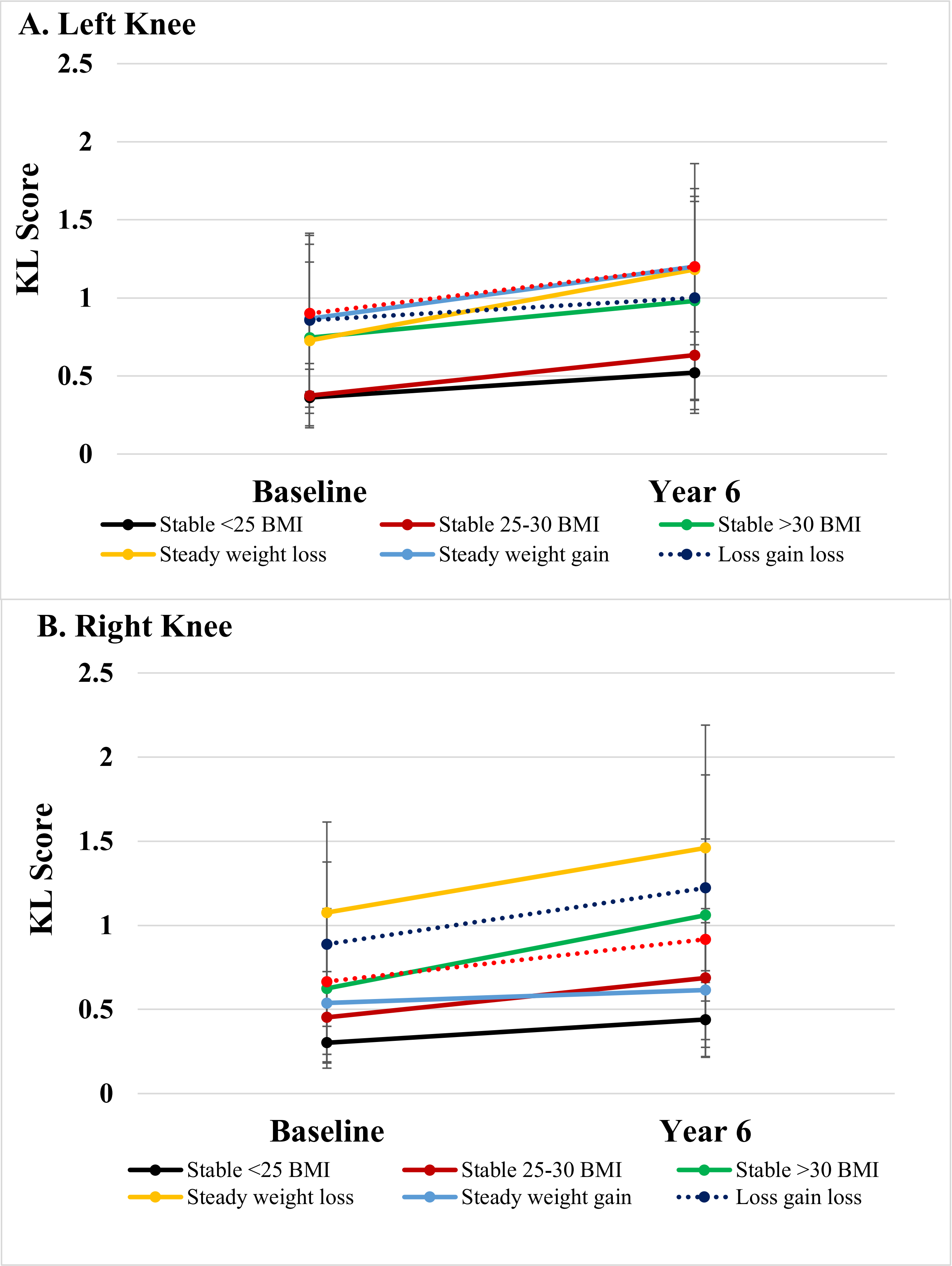

Supplemental Figure 1. Kellgren Lawrence (KL) scores at baseline and month 72. Values are means ± SD.
