# Supplementary material for: Weight-cycling over 6 years is associated with pain, physical function and depression in the Osteoarthritis Initiative cohort
Source: Sci Rep. 2023 Oct 9;13:17045. doi: 10.1038/s41598-023-44052-3 (PMC10562481; doi:10.1038/s41598-023-44052-3)
Supplement: Supplementary file 2 — Supplementary Table 1. [file 41598_2023_44052_MOESM2_ESM.docx]

**Supplemental Table 1.** Knee pain severity and prevalence of participants who became pain free or developed pain from pan free status from baseline to month 72. for among groups with different patterns of weight-BMI change. Values are expressed as means ± SD or in percent.

Stable weight Stable weight Stable weight Steady Steady Cycling Cycling

<25 kg/m^2^ 25-29.9 kg/m^2^ >30 kg/m^2^ weight loss weight gain loss gain loss gain loss gain

(n) (220) (206) (97) (24) (24) (85) (75) p across

groups

**Left limb**

Pain severity

Baseline 1.7 ± 2.3 2.1 ± 2.4 2.8 ± 2.7 3.9 ± 2.8 3.0 ± 3.1 3.6 ± 3.2 2.7 ± 2.8 <.001

Month 72 1.9 ± 2.3 2.0 ± 2.5 2.3 ± 2.5 4.2 ± 3.3 3.9 ± 3.0 3.1 ± 2.9 3.2 ± 2.9 <.001

Pain change

Pain to pain free 27.2 33.3 30.3 16.6 0.0 20.3 23.2 .164

Pain free to pain 37.2 34.7 40.0 50.0 55.5 56.0 60.0 <.0001

**Right limb**

Pain severity

Baseline 1.7 ± 2.2 2.1 ± 2.3 2.5 ± 2.6 3.4 ± 2.8 2.2 ± 2.3 3.0 ± 2.9 2.7 ± 2.7 <.001

Month 72 2.1 ± 2.6 2.1 ± 2.3 2.7 ± 2.8 2.9 ± 2.7 3.7 ± 2.8 2.6 ± 2.6 2.6 ± 2.5 .012

Pain change

Pain to pain free 22.5 27.4 23.3 11.7 14.2 20.7 15.2 .707

Pain free to pain 37.6 47.2 52.8 42.9 80.0 51.6 40.7 .027
